# Seroepidemiology of *Toxoplasma gondii* infection in drivers involved in road traffic accidents in the metropolitan area of Guadalajara, Jalisco, Mexico

**DOI:** 10.1186/1756-3305-6-294

**Published:** 2013-10-11

**Authors:** Ma de la Luz Galván-Ramírez, Laura Verónica Sánchez-Orozco, Laura Rocío Rodríguez, Saúl Rodríguez, Enrique Roig-Melo, Rogelio Troyo Sanromán, Erwin Chiquete, Juan Armendáriz-Borunda

**Affiliations:** 1 Department of Physiology, Neurophysiology Laboratory, University Center of Health Sciences, University of Guadalajara, Guadalajara, Jalisco, Mexico; 2 Institute of Molecular Biology in Medicine and Gene Therapy, University Center of Health Sciences, University of Guadalajara, Guadalajara, Jalisco, Mexico; 3 Institute of Ophthalmology and Visual Sciences, University Center of Health Sciences, University of Guadalajara, Guadalajara, Jalisco, Mexico; 4 Department of Neurology and Psychiatry, The Salvador Zubirán National Institute of Medical Sciences and Nutrition, Mexico, D.F, Mexico

**Keywords:** Toxoplasmosis, Diagnosis, Traffic accidents, ELISA, Zoonoses

## Abstract

**Background:**

The prevalence of toxoplasmosis in the general population of Guadalajara, Mexico, is around 32%. Toxoplasmosis can cause ocular lesions and slowing of reaction reflexes. Latent toxoplasmosis has been related with traffic accidents. We aimed to assess the prevalence of anti-*Toxoplasma gondii* antibodies and visual impairments related with traffic accidents in drivers from the metropolitan Guadalajara.

**Methods:**

We prospectively evaluated the prevalence of IgG and IgM anti-*T. gondii* antibodies in 159 individuals involved in traffic accidents, and in 164 control drivers never involved in accidents. Cases of toxoplasmosis reactivation or acute infection were detected by PCR in a subset of 71 drivers studied for the presence of *T. gondii* DNA in blood samples. Ophthalmologic examinations were performed in drivers with IgG anti-*T. gondii* antibodies in search of ocular toxoplasmosis.

**Results:**

Fifty-four (34%) traffic accident drivers and 59 (36%) controls were positive to IgG anti-*T. gondii* antibodies (*p* = 0.70). Among the 113 seropositive participants, mean anti-*T. gondii* IgG antibodies titers were higher in traffic accident drivers than in controls (237.9 ± 308.5 IU/ml vs. 122.9 ± 112.7 IU/ml, respectively; *p* = 0.01 by Student’s t test, *p* = 0.037 by Mann–Whitney U test). In multivariate analyses, anti-*T. gondii* IgG antibody titers were consistently associated with an increased risk of traffic accidents, whereas age showed an inverse association. The presence of IgM-anti-*T. gondii* antibodies was found in three (1.9%) subjects among traffic accident drives, and in two (1.2%) controls. Three (4.2%) samples were positive for the presence of *T. gondii* DNA, all among seropositive individuals. No signs of ocular toxoplasmosis were found in the entire cohort. Moreover, no other ocular conditions were found to be associated with the risk of traffic accidents in a multivariate analysis.

**Conclusions:**

Anti-*T. gondii* antibody titers are associated with the risk of traffic accidents. We could not determine any association of ocular toxoplasmosis with traffic accidents. Our results warrant further analyses in order to clarify the link between toxoplasmosis and traffic accidents.

## Background

Toxoplasmosis is a worldwide disease caused by an obligate intracellular protozoan named *Toxoplasma gondii*. Infection is commonly acquired by consumption of raw or undercooked meat particularly pork and lamb containing cysts with bradyzoites, as well as fruits and vegetables contaminated with oocysts. Also, the infection can pass into the newborn by congenital transmission from pregnant primo-infected women. People can get it from blood transfusion, organ transplantation, and it can also pass to butchers or personnel that handle products infected with the parasite and accidental exposure with contaminated blood [1,2].

Human toxoplasmosis is asymptomatic in 80% of the population, as tissue cysts can persist indefinitely during the host’s life. If an individual becomes immunocompromised, these tissue cysts serve as reservoir from which disseminated or local infection can develop [3], particularly in individuals with HIV/AIDS [4], *T. gondii* can cause lethal encephalitis, myocarditis, pneumonitis and chorioretinitis. Personality changes have been reported in infected people from the Czech Republic [5,6]. Additionally, slow reaction times and impaired motor performance have been reported in subjects with latent infection [7,8]. The high levels of dopamine could explain the behavioral changes, since *T. gondii* has genes that codify for aromatic amino acid hydroxylase enzymes, which metabolize phenylalanine to tyrosine and tyrosine to L-DOPA, the precursor of dopamine. It is hypothesized that increased dopamine levels cause changes in neurotransmitters and as a consequence, in behaviour [9,10]. Latent toxoplasmosis has been associated with schizophrenia [3]. In Mexico, prevalence of anti-*Toxoplasma* antibodies has been reported to be 37.24% in mentally ill patients, 18.26% higher than the general population [4].

Epidemiologic reports of seroprevalence indicate that one third of the world population has been in contact with the parasite [11]. The mean National seroprevalence reported in Mexico by 1992 was 50%, and in the State of Jalisco it was found to be 32% [12]. Recently, a meta-analysis reported a weighted mean prevalence of 20.26% in the Mexican general population [4].

The Ministry of Roads and Transport in Jalisco, reported 53,485 traffic accidents during 2008 in the metropolitan area of Guadalajara, Jalisco; during the last five years, the Jalisco Health Ministry reported motor accidents as the leading cause of death in the age group 15 to 19 years, and the second leading cause in individuals aged 20 to 50 years. Since *T. gondii* infection can cause chorioretinitis, longer reaction times and impaired motor performance, the purpose of this study was to determine the prevalence of IgM and IgG anti-*T. gondii* antibodies in drivers who had accidents in the metropolitan Guadalajara. We also aimed to identify visual impairments and the risk factors associated with *T. gondii* seropositivity in this population, as compared with a control group.

## Methods

### Ethical aspects

The purpose and procedures of the study were explained to all participants, and a written informed consent was obtained from all of them. The Institutional Ethical Committee from The University Center of Health Sciences, University of Guadalajara, approved this study.

### Study population with traffic accidents

This group included 121 men and 38 women (mean age 37.09 ± 11.8 years). The drivers were sent for medical attention to the Delgadillo Araujo Green Cross Hospital. All drivers included in the study presented with injuries, and an exclusion criterion was a positive alcoholmeter test.

### Study population not involved in traffic accidents

This group consisted of 164 drivers including 124 men and 40 women (mean age 39.68 ± 11.8 years). All of them were residents from Guadalajara. They had never been involved in traffic accidents, and were randomly selected and invited to participate when they visited the Ministry of Road and Transit in Guadalajara. These drivers were considered controls for comparisons.

### Sample and data collection

Blood samples were collected from all drivers from May to December 2011. Sociodemographic, clinical, and risk factor information related with *T. gondii* infection were explored through a structured questionnaire in all participants. Cats at home, raw or undercooked meat consumption, raw fruit and vegetable consumption, blood transfusion and organ transplantation were investigated. Clinical data included the presence of underlying visual impairments (astigmatism, hypermetropia, presbyopia, myopia).

### Serological test for *T. gondii* antibodies

Serum samples were obtained by centrifugation of fresh whole blood from all participants. Blood samples were sent to the Laboratory of Neurophysiology in University Center of Health Sciences, University of Guadalajara. Serum samples were kept frozen at −20°C until they were processed. IgG and IgM antibodies to *T. gondii* were tested in all samples by commercially available enzyme immunoassay (ELISA); Toxo IgM of capture, and Toxo IgG indirect detection of antibody (Platelia TM Toxo, Bio-Rad, Marnes-la-Coquette France). Plates were read at 450/620 nm and optical density values were plotted in a standard curve for IU/ml. Titers < 6 UI/Ml were considered negative, 6 to 9 indeterminate, and > 9 to 200 positive for latent infection. Samples with values > 200 were checked out twice. All tests were performed following the instructions of the manufacturer.

### Detection of *T. gondii* DNA from blood samples

Genomic DNA was obtained from lymphocytes. Briefly, 5 ml of anti-coagulated blood was used to isolate the lymphocyte population using Lymphoprep™ (Axis-Shield). Then, a phenol-chloroform DNA extraction was performed. *T. gondii* DNA detection was analyzed by PCR amplification of a 112 bp fragment from *T. gondii* repetitive DNA sequence (REP) of 529 pb, followed by a semi-nested PCR amplification of a 72 bp fragment. The primers for the first PCR were: REPS1, 5′-AGAGACACCGGAATGCGATCT and REPAS, 5′-TTCGTCCAAGCCTCCGACT and REPS2, 5′-TCGTGGTGATGGCGGAGAGAATTGA with REPAS for the second PCR. Also, a second semi-nested PCR was developed to identify a fragment corresponding to B1 gene of *T. gondii*, with the following primers: B1S: 5′- GAAAGCCATGAGGCACTCCA, B2S: 5′– CGAGTAGCACCTGAGGAGAT and B1AS: 5′-TTCACCCGGACCGTTTAGC. DNA used as positive control was obtained from *T. gondii* cultures (Tachyzoites RH strain). Appropriate measures were taken to minimize the risk of sample cross-contamination. These measures comprised the inclusion of samples from normal subjects and aliquots of water as negative controls. Positive results were considered after detection of *T. gondii* DNA in two independent assays.

### Diagnosis of ophthalmic diseases

A comprehensive ophthalmologic examination as well as detailed retinal examination were performed in patients positive to *T. gondii* antibodies in search of any ocular manifestation of toxoplasmosis, such as vitreous inflammation, chorioretinal scar, retinichoroiditis active lesion, anterior or posterior uveitis, retinal vasculitis, or neuroretinitis [13].

### Statistical analysis

The statistical analyses were performed by using Epi Info (v. 3.3.2) and SPSS (v. 18) statistical packages. Type I error was set at *p* < 0.05. Categorical variables are expressed as relative frequencies (percentages) and continuous variables as means ± standard deviations (SD) or medians with interquartile range, if distributed normally or non-parametrically, respectively. We used the chi-square test and the Fisher exact test for comparison of frequencies among groups. Odds ratio (OR) with 95% confidence interval (CI) was used to estimate the magnitude of an association with a binary outcome. Student’s t test was used to compare continuous variables normally distributed between two groups, and Mann–Whitney *U* test was performed to compare continuous non-parametric variables between two groups. Given that an unexpectedly low frequency of seropositivity to anti-*T. gondii* IgG antibodies was found among the age stratum 40–49 years in traffic accident drivers, a generalized linear model was used to assess the interaction among age strata and toxoplasmosis seropositivity, either as categorical or continuous (antibodies titers) variable. To find independent predictors of a binary outcome (traffic accidents, toxoplasmosis seropositivity and ocular findings), we constructed multivariate analyses by forward stepwise logistic regression. Adjusted ORs with 95% CIs are provided. A *p* <0.05 was considered statistically significant in most final multivariate models. However, a Bonferroni correction of the *p* value (setting *p* < 0.025 as the new statistical inference limit) was used in a second logistic regression model after excluding the age stratum 40–49 years to evaluate whether the risk of traffic accidents is modified after excluding this age group behaving exceptionally with respect to seropositive prevalence.

## Results

We studied 323 participants (159 traffic accident drivers and 164 control drivers). The median age was 37 years (35 years in traffic accident drivers vs. 39 years in control drivers, (*p* = 0.049), with 75.9% men (76.1% in traffic accident drivers vs. 75.6% in control drivers, (*p* = 0.92). Most individuals were married (62.8% of participants; 62.9% in traffic accident drivers vs. 62.8% in control drivers, (*p* = 0.98) and with >9 years of formal education (77% of participants; 74.7% in traffic accident drivers vs. 79.3% in control drivers, (*p* = 0.33).

### Frequency of IgG and IgM anti-*T. gondii* antibodies

**Figure 1:**
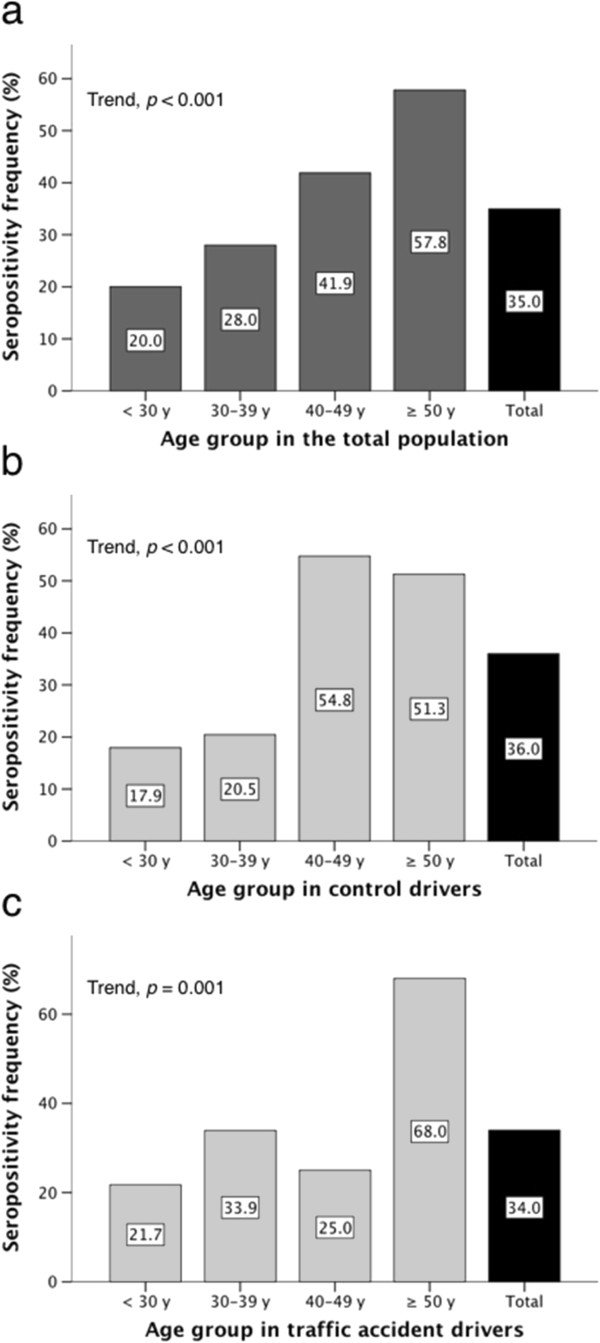
**Frequency of anti-*T. gondii *IgG antibodies in serum of the participants, according to age. *a***: the total cohort. ***b***: control drivers, ***c***: traffic accident drivers.

**Figure 2:**
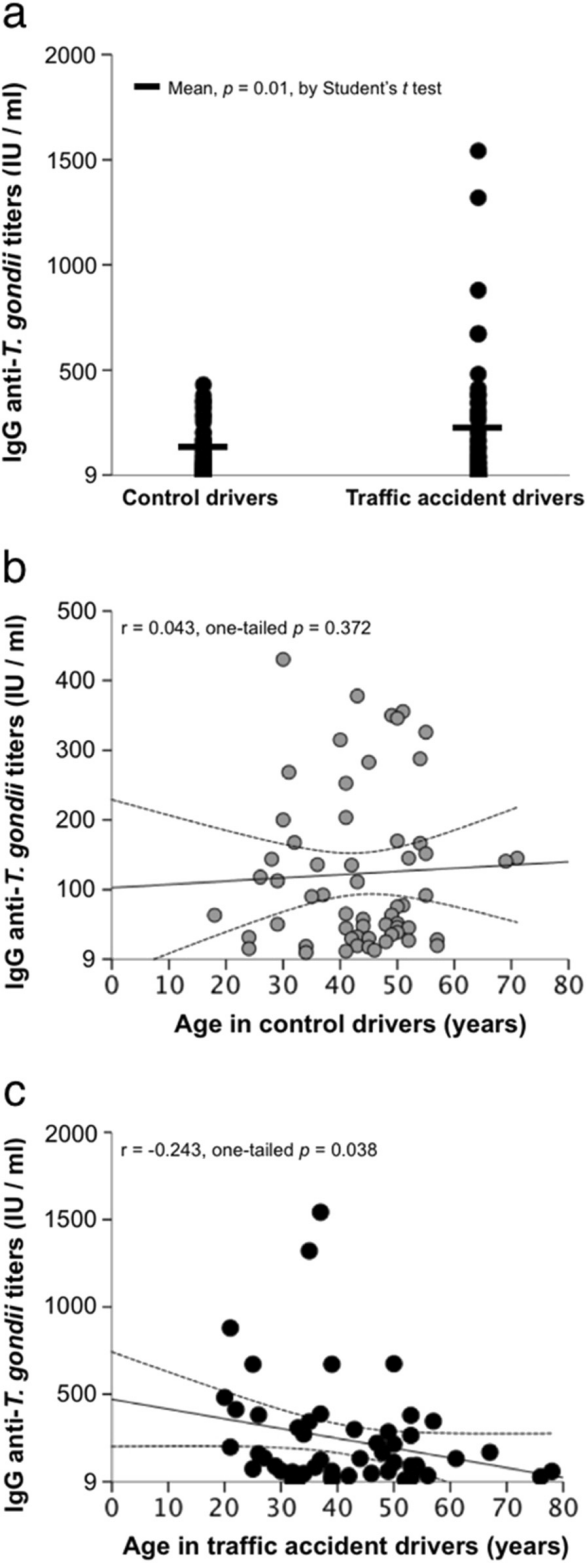
**Anti-*T. gondii *IgG antibodies titers. *a***: antibody titers according to study group. ***b***: correlation of antibody titers with age among control drivers. ***c***: correlation of antibody titers with age among traffic accident drivers.

Fifty-four out of 159 (34%) traffic accident participants and 59 of 164 (36%) controls were positive to anti-*T. gondii* IgG antibodies (*p* = 0.70). Among the 113 seropositive participants mean anti-*T. gondii* IgG antibodies titers were higher in traffic accident drivers than in controls (237.9 ± 308.5 IU/ml vs. 122.9 ± 112.7 IU/ml, respectively; *p* = 0.01 by Student’s t test, *p* = 0.037 by Mann–Whitney U test). Seropositive status increased with age (Figure 1); however, this tendency was not consistent in the traffic accidents group, as the age group 40–49 years showed a relatively low seroprevalence. This phenomenon partly explained a negative correlation between age and anti-*T. gondii* IgG antibodies titers in the traffic accidents group, which was not observed among controls (Figure 2). As a consequence, univariate ORs for the association of several categorical variables with traffic accidents showed an aberrantly protecting effect in the age group 40–49 year (Table 1).

**Table 1:** **Univariate ORs on factors associated with IgG anti-*T. gondii *seropositivity**

**Subset**	**Traffic accident drivers**	**Control drivers**	**Odds ratio**	**95% CI**	***p *value**
	**seropositive / subtotal (%)**	**seropositive / subtotal (%)**			
Age strata					
20-29 y	10/ 46 (21.7%)	7 / 39 (17.9%)	1.27	0.38 – 4.25	0.66
30-39 y	19 / 56 (33.9%)	9 / 44 (20.4%)	2.00	0.73 – 5.55	0.14
40-49 y	8 / 32 (25%)	23 / 42 (54.8%)	0.28	0.09 – 0.83	0.01*
50-59 y	17 / 25 (68%)	21 / 39 (53.8%)	1.82	0.57 – 5.96	0.26
Age strata in men					
Men < 30 y	9 / 40 (22.5%)	5 / 33 (15.2%)	1.63	0.49 – 4.43	0.51
Men 30–39 y	13 / 37 (35.1%)	8 / 29 (27.6%)	1.42	0.49 – 4.09	0.09
Men 40–49 y	8 / 26 (30.8%)	18 / 34 (52.9%)	0.39	0.13 – 1.15	0.87
Men > 50 y	12 / 18 (66.7%)	18 / 28 (64.3%)	1.11	0.32 – 3.87	0.38
Age strata in women					
Women < 30 y	1 / 6 (16.7%)	2 / 6 (33.3%)	0.40	0.03 – 6.17	0.07
Women 30–39 y	6 / 19 (31.6%)	1 / 15 (6.7%)	6.46	0.68 – 61.70	0.28
Women 40–49 y	0 /6 (0%)	5 / 8 (62.5%)	0.33	0.13 – 8.40	0.09
Women >50 y	5 / 7 (71.5%)	3 / 11 (27.3%)	6.67	0.81 – 54.9	0.69
Educational level					
1-6 y	2 / 12 (16.6%)	3 / 7 (42.9%)	0.47	0.04 – 5.01	0.46
7–12 y	28 / 72 (38.9%)	25 / 62 (40.3%)	0.94	0.44 – 2.0	0.86
>12 y	24 / 75 (32.0%)	32 / 95 (33.7%)	0.93	0.46 – 1.86	0.82
Socioeconomic level					
Low	10 / 24 (41.7%)	14 / 32 (44.7%)	0.92	0.27 – 3.06	0.88
Medium	28 / 86 (32.6%)	22 / 70 (31.4%)	1.05	0.51 – 2.19	0.88
High	16 / 49 (32.7%)	24 / 62 (38.7%)	0.77	0.32 – 1.81	0.51

IgG antibody titers were further classified by class intervals with the following ranges 9–49.99, 50–99.99, 100–149.99, 150–199.99 and 200 or more IU/ml. The results showed a positive non-significant association with the risk for traffic accidents; however, this tendency was not uniform across the age strata. Then, a generalized linear model was constructed to analyze interactions between age and anti-*T. gondii* IgG antibodies titers among the whole cohort and among study groups separately. This model did not show a significant age-antibody titer interaction; nevertheless, logistic regression models constructed to evaluate the association of independent variables with traffic accidents showed a consistent significant association between anti-*T. gondii* IgG antibody titer with the risk of traffic accidents, even after the exclusion of the age group 40–49 years (Table 2). Hence, anti-*T. gondii* IgG antibodies titers were consistently associated with an increased risk of traffic accidents, whereas age showed an inverse association.

**Table 2:** Multivariate analyses on the risk of traffic accidents: two forward stepwise binary logistic regression models*

**Subset**	**Beta**	**SE**	**Wald**	**df**	**OR**	**95% CI**	***p *value**
**Model 1: All participants (n** = **323)**							
Age	−0.242	0.107	5.090	1	0.785	0.636 – 0.969	0.024
IgG anti-*T. gondii* titers	0.002	0.001	3.992	1	1.002	1.000 – 1.004	0.046
Constant	0.425	0.275	2.379	1	1.529		0.123
**Model 2: Excluding the age group 40 – 49 years (n** = 249) **							
Age	−0.244	0.115	4.477	1	0.783	0.625 – 0.982	0.034
IgG anti-*T. gondii* titers	0.003	0.001	5.107	1	1.003	1.000 – 1.005	0.024
Constant	0.409	0.277	2.180	1	1.505		0.140

CI = Confidence interval, df = Degrees of freedom, OR = Odds ratio, SE = Standard error.

* Adjusted for sex, marital status, educational level and economical activity.

** A second multivariate model was constructed after excluding the age stratum 40–49 years (n = 74), due to an unexpected low frequency of seropositive cases in this group. The second model crossed the new *p* value of 0.025 after Bonferroni correction.

The presence of IgM-anti-*T. gondii* antibodies was found in only three (1.9%) subjects among traffic accident drives, and in two (1.2%) control drivers (*p* = 0.673). As expected, the five IgM seropositive individuals exhibited higher anti-*T. gondii* IgG antibodies titers, as compared with IgM seronegative persons (318.5 ± 250.1 IU/ml vs. 60.14 ± 158.9 IU/ml, respectively; *p* < 0.001).

### Risk factors for *T. gondii* infection

Common risk factors for *T. gondii* infection were analyzed for their association with the seropositive status. Neither univariate nor adjusted multivariate analyses showed a significant association of these traditional *Toxoplasma* risk factors with seropositivity in our cohort (Table 3).

**Table 3:** Risk factors associated with *T. gondii* infection

	**No. of subjects tested**	**Prevalence of *T. gondii *infection**	***p *value**	**Crude OR**	**95% CI**	**Adjusted OR**	**95% CI**	***p *value**
		**No.%**						
**Cats at home**								
Yes	116	46 39.7	0.209	1.35	0.840 – 2.172	1.372	0.473 – 3.981	0.560
No	205	67 32.7						
**Raw meat consumption**								
Yes	49	18 36.7	0.772	1.09	0.581 – 2.060	1.790	0.689 – 4.680	0.230
No	266	92 34.5						
**Undercooked meat consumption**								
Yes	106	34 32.1	0.477	0.83	0.503 – 1.371	0.680	0.355 – 1.304	0.244
No	202	73 36.1						
**Raw fruits and vegetables consumption**								
Yes	272	95 34.9	0.948	0.97	0.513 – 1.860	0.569	0.220 – 1.460	0.243
No	48	17 35.2						
**Blood transfusion**								
Yes	20	11 55	0.054	2.3	0.964 – 5.956	2.503	0.825 – 7.591	0.105
No	302	102 33.8						

CI = Confidence interval, OR = Odds ratio.

### *T. gondii* DNA detection

A total of 71 subjects underwent an additional venopuncture to obtain blood samples for genomic DNA: 33 and 38 from the traffic accidents and control group, respectively. Three (4.2%) samples were positive for the presence of *T. gondii* DNA in REP and only one of these was positive for B1 gene. Two out of three positive samples were from the traffic accidents group and one pertained to the control group. Among these samples, one was positive to both IgM and IgG anti-*T. gondii* antibodies and two were only positive to IgG antibodies. None of the DNA-positive samples were negative to IgM or IgG anti-*T. gondii* antibodies. Nevertheless, only one sample was positive to *Toxoplasma* DNA using B1 gene (Table 4).

**Table 4:** Detection of DNA *Toxoplasma gondii *and antibodies in traffic accidents and controls drivers*

**Sample N°. / group**	**REP**	***B1 *gene**	**IgM ELISA**	**IgG ELISA**	**IgG WB**
# 113 / Control group	Positive	Negative	Negative	Positive	Positive
# 46 / Traffic accidents group	Positive	Negative	Negative	Positive	Positive
# 62 / Traffic accidents group	Positive	Positive	Positive	Positive	Positive

* Seventy-one samples previously positive for IgG anti-*T. gondii* antibodies were tested for *Toxoplasma* DNA in the traffic accidents and control groups. ELISA = Enzyme-linked immunosorbent assay, WB = western-blot, REP = repetitive sequence of *Toxoplasma gondii*.

### Ocular findings

A structured questionnaire was applied to 278 participants (137 traffic accidents drivers and 141 controls) in order to examine the association of any ophthalmologic abnormality (including possible refraction errors) with traffic accidents. Ocular findings in drivers involved in accidents were found in 81 out of 137 (59.1%) and in 100 out of 141 (70.9%) control drivers. Logistic regression was performed for ocular findings and traffic accidents as dependent variable, adjusting for age, gender and socioeconomic level. No statistical association was found with any ocular finding analyzed (Table 5). Moreover, a detailed ophthalmologic examination was performed in 75 of 114 seropositive drivers in order to determine whether ocular toxoplasmosis explained the link between seropositivity to anti-*T. gondii* antibodies and traffic accidents. None of the participants in either study group showed any ocular injury related with toxoplasmosis (i.e., vitreous inflammation, chorioretinal scar, retinochoroiditis active lesion, anterior or posterior uveitis, retinal vasculitis, or neuroretinitis).

**Table 5:** **Ocular findings and anti-*T. gondii *IgG antibodies**

**Ocular condition**	**Crude OR**	**95% CI**	***p value***	**Adjusted OR**	**95% CI**	***p value***
Astigmatism	0.520	0.116 – 2.322	0.388	1.23	0.231 – 6.60	0.804
Hypermetropia	-	-	-	-	-	-
Presbyopia	0.643	0.242 – 1.710	0.375	1.13	0.224 – 5.720	0.880
Myopia	0.278	0.027 – 2.903	0.266	0.877	0.146 – 5.27	0.886
Glaucoma	-	-	-	-	-	-
Others	4.50	0.337 – 60.154	0.237	3.339	0.499 – 22.324	0.214
Two or more	0.857	0.191 – 3.853	0.841	1.373	0.219 – 8.615	0.735
No problem	2.117	0.784 – 5.716	0.134	0.403	0.129 – 1.259	0.118

## Discussion

We found that anti-*T. gondii* IgG antibodies titers are associated with the risk of traffic accidents occurring in metropolitan Guadalajara, Mexico. The fact that seropositive status as a categorical variable was not associated with the risk of traffic accidents, but this association was evident when considering IgG antibodies as a continuous variable, suggests that high antibody levels could be a marker of a subclinical reactivation and/or acute infection. This hypothesis is in partly supported by the finding that high IgG levels occurred in participants who were positive to anti-*T. gondii* IgM antibodies. Furthermore, *T. gondii* DNA was found only in seropositive individuals (although only one DNA-positive participant was also positive to IgM antibodies).

Our findings support previous observations in this new but growing research area [14-16]. Taken together, this study and previous findings suggests that the increased risk of traffic accidents could possibly be the consequence of acute toxoplasmosis, rather than the increasing effect of latent toxoplasmosis, a hypothesis previously proposed by studies conducted in the Czech Republic and Turkey [14-16].

In this study, no statistical differences were found in the frequency of recent toxoplasmosis infection determined by IgM anti-*T. gondii* antibodies in drivers involved in traffic accidents and control group since the presence of these antibodies was detected in 3 (1.9%) and 2 (1.2%) respectively. Similar results were found in normal Mexican pregnant women 1/50 (2.0%) determined by ELISA [17]. From the three positive samples to IgM anti- *T. gondii* antibodies only two were recovered by PCR; and only in one of these samples *T. gondii* DNA was positive for the region REP and B1 gene. It has been reported that in some cases the positive values to IgM persist for long periods of time, this could be the reason for not detecting *Toxoplasma* DNA in the other blood sample positive to IgM anti-*Toxoplasma* antibodies. Two more samples were positive to *Toxoplasma* DNA only in the REP region; both samples were positive to IgG anti-*Toxoplasma* antibodies. Previous studies have reported the presence of *Toxoplasma* DNA in the absence of anti-*Toxoplasma* antibodies [18]. In our study, the sensitivity for DNA detection was better using the REP region of *Toxoplasma gondii* genome as a target compared to B1 gene, the same results have been reported previously [19-21]. Since nested PCR detects DNA directly, this method can contribute to increased sensitivity for the detection of reactivation of *Toxoplasma* infection, especially in areas where the prevalence of this disease is high.

Prevalence of IgG *anti-T. gondii* antibodies increases with age in both groups, similar results have been reported in blood donors where *anti-T. gondii* antibody prevalence increased after the age of 20 years [4].

In relation to the presence of anti-*Toxoplasma* antibodies and gender, in this study population, the percentage of males positive to these antibodies was higher than females; even if, no statistical differences were found. These results are similar to those previously reported by Hye-Jin Ahn, et al. 2012, where positive rate was significantly different between genders; 20.6% for male and 13.1% for female (*P* < 0.05) [22].

The educational level and socioeconomic strata are interrelated variables; in this study population no statistical significant difference was found between the presence of anti-*T. gondii* antibodies and demographic data. Similar results were reported in the same geographic area [4] and in the state of Durango, Mexico [23]. Nevertheless, a trend for a risk to be involved in traffic accidents was found in anti-*Toxoplasma* positive drivers with low and medium socioeconomic level compared with high socioeconomic level, similar results were found in Mexican populations of Durango state in subjects with workplace accidents who showed statistical significance with the presence of anti-*Toxoplasma* antibodies, low socioeconomic level and workplace accidents [24].

Risk factors associated with *Toxoplasma* infection such as: presence of cats at home, raw and/or undercooked meat consumption, and raw vegetables consumption, were not associated to the presence of anti-*T. gondii* antibodies in this study population. Probably, another risk factor, such as contaminated water might explain the high prevalence of anti-*Toxoplasma* antibodies in this group. This risk factor was not considered in this study [25].

In relation to blood transfusion, the prevalence of *Toxoplasma gondii* was higher in drivers who had previously received a blood transfusion 55% (11/20) compared with drivers who had never received a transfusion 33.8% (102/302). Statistical analysis between both groups are close to the significance (p = 0.054). Blood transfusion has been previously reported as a risk factor of *Toxoplasma infection*[2].

Furthermore, no statistical differences were found in drivers with latent infection (presence of IgG-anti-*T.gondii* antibodies) between both groups, since the prevalence of these antibodies were very close in traffic accidents and control group drivers, opposing results were reported in Czech populations where an antibody prevalence of 39.7% was found in victims of traffic accidents compared to18% in controls [15]. Also, in Turkey population significant differences were found in subjects involved in traffic accidents compared with control group drivers (IgG anti-*Toxoplasma* antibodies 24.32% vs 6.48%) [26].

Relating to ocular findings determined by the questionnaire, presence of abnormalities was found in both groups in this study; astigmatism, presbyopia, myopia and glaucoma were more frequent in the control group. The study of ocular fundus performed in anti-*Toxoplasma* seropositive drivers with this diagnosis, did not show any ocular injury related to toxoplasmosis. In immunocompetent subjects, the signs and symptoms of ocular findings due to *Toxoplasma* are a consequence of the inflammatory immune response rather than the parasite itself. In our study, all subjects positive to IgG anti-*Toxoplasma* antibodies did not have any sign or symptom associated with ocular toxoplasmosis. Probably, *Toxoplasma* strains affecting our population are different from the virulent *Toxoplasma* type I; since this strain has been associated with ocular toxoplasmosis in healthy Brazilian people [27,28].

Finally, *Toxoplasma* infection can be asymptomatic in 80% of immune-competent hosts in latent stage; cysts can remain in brain and muscles for a lifespan in the host. On the other hand, motor functions can be diminished representing an important factor in traffic accidents in human infection. In this study, we were not able to explain whether or not longer reaction times and impaired motor performance were factors associated to traffic accidents or *Toxoplasma* infection in drivers included in this study; since we did not evaluate these parameters. Future studies must be performed in order to investigate whether neurological damage caused by *Toxoplasma* infection affects motor reflexes in drivers.

## Conclusions

In this study, considering IgG anti-*Toxoplasma* antibody-positive drivers, the titers are significantly higher in the traffic accident group compared to the control group, whereas age showed an inverse association. No ocular findings were associated to ocular toxoplasmosis.

## Competing interests

The authors declare that they have no competing interests.

## Authors’ contributions

MLG conceived and designed the research, LVSO carried out Diagnostic tests through PCR, RT and EC performed the statistical analyses, MLG, LVSO and EC wrote the manuscript, JAB reviewed the manuscript, ER conducted a diagnostic fundus study and LRR conducted Diagnostic by means of ELISA. All authors read and approved the final version of the manuscript.
